# Chronic fatigue syndrome (CFS) or myalgic encephalomyelitis (ME) is different in children compared to in adults: a study of UK and Dutch clinical cohorts

**DOI:** 10.1136/bmjopen-2015-008830

**Published:** 2015-10-28

**Authors:** Simon M Collin, Roberto Nuevo, Elise M van de Putte, Sanne L Nijhof, Esther Crawley

**Affiliations:** 1 Centre for Child & Adolescent Health, School of Social and Community Medicine, University of Bristol, Bristol, UK; 2 Department of Paediatrics, Wilhelmina Children's Hospital, University Medical Centre, Utrecht, The Netherlands

**Keywords:** EPIDEMIOLOGY, PAEDIATRICS

## Abstract

**Objective:**

To investigate differences between young children, adolescents and adults with chronic fatigue syndrome/myalgic encephalomyelitis (CFS/ME).

**Study design:**

Comparison of clinical cohorts from 8 paediatric and 27 adult CFS/ME services in the UK and a paediatric randomised controlled trial from the Netherlands. Outcome measures include: fatigue (the UK—Chalder Fatigue Scale); Disability (the UK—SF-36 physical function subscale; the Netherlands—CHQ-CF87); school attendance, pain, anxiety and depression (the UK—Hospital Anxiety & Depression Scale, Spence Children's Anxiety Scale; the Netherlands—Spielberger State-Trait Anxiety Inventory for Children, Children's Depression Inventory); symptoms; time-to-assessment; and body mass index. We used multinomial regression to compare younger (aged <12 years) and older (aged 12–18 years) children with adults, and logistic regression to compare UK and Dutch adolescents.

**Results:**

Younger children had a more equal gender balance compared to adolescents and adults. Adults had more disability and fatigue, and had been ill for longer. Younger children were less likely to have cognitive symptoms (OR 0.18 (95% CI 0.13 to 0.25)) and more likely to present with a sore throat (OR 1.42 (1.07 to 1.90). Adolescents were more likely to have headaches (81.1%, OR 1.56 (1.36% to 1.80%)) and less likely to have tender lymph nodes, palpitations, dizziness, general malaise and pain, compared to adults. Adolescents were more likely to have comorbid depression (OR 1.51 (1.33 to 1.72)) and less likely to have anxiety (OR 0.46 (0.41 to 0.53)) compared to adults.

**Conclusions:**

Paediatricians need to recognise that children with CFS/ME present differently from adults. Whether these differences reflect an underlying aetiopathology requires further investigation.

**Trial registration numbers:**

FITNET trial registration numbers are ISRCTN59878666 and NCT00893438. This paper includes secondary (post-results) analysis of data from this trial, but are unrelated to trial outcomes.

**Strengths and limitations of this study**

- This study benefited from large sample sizes, and data on chronic fatigue syndrome/myalgic encephalomyelitis (CFS/ME) symptoms and comorbid mood disorders were collected in the same way from UK children and adults.
- Adults and children were recruited from specialist CFS/ME services in the UK and the Netherlands, so the results may not be generalisable to other settings, such as primary care.
- Fatigue, disability, pain and mood, were measured differently in the UK and the Netherlands, making it difficult to compare these characteristics directly.

## Introduction

The incidence of chronic fatigue syndrome/myalgic encephalomyelitis (CFS/ME) has two distinct age peaks, at 10–19 years and 30–39 years.^1^ Little is known about the underlying aetiopathology of CFS/ME and whether it differs between children and adults. Studies investigating heterogeneity (phenotypes) in adult^2–5^ and paediatric^6^ CFS/ME patients have described some similarities in the delineation of paediatric and adult CFS/ME phenotypes, based mainly on the presence/absence of musculoskeletal pain and ‘acute’ illness symptoms (swollen glands, sore throat, headache). However, prognosis is much better in children than in adults, with 54–94% of children improving or making a complete recovery from the illness,^7^ ^8^ compared with a recovery rate of not more than 22% in adults.^9^

No previous study has compared the clinical features of paediatric and adult CFS/ME patients, which means that clinical guidelines, teaching and definitions for paediatric patients are based on adult descriptions of CFS/ME. Paediatricians also need more information about age-related differences between younger and older (adolescent) children. We previously compared 32 primary school-aged children with adolescents, which showed that they had similar symptoms at presentation.^10^ However, the sample size was too small to allow detailed comparison between the two age groups.

In this study, we used three large clinical cohorts to compare the characteristics of CFS/ME in children, adolescents and adults. We hypothesised that CFS/ME in adults would present differently in children compared to adults, but that younger children would be similar to older children. We also compared adolescent patients diagnosed with CFS/ME in the UK with adolescent patients recruited into a large randomised controlled CFS/ME treatment trial in the Netherlands,^8^ to investigate whether paediatric CFS/ME phenotypes might be robust across different countries and healthcare settings, as has been demonstrated for adult patients with CFS/ME.^11^

## Methods

### UK adult and paediatric clinical cohorts

UK data were collected from all NHS specialist services (see online supplementary material) that participated in the CFS/ME National Outcomes Database between August 2004 and October 2014. During this period, clinical assessment data and patient-reported measures were collected for approximately 10 000 adults and 1500 children and young people, from 27 adult and 8 paediatric CFS/ME services. Patients were included if they were given a diagnosis of CFS/ME in accordance with Centers for Disease Control and Prevention (CDC) criteria (to 2007)^12^ ^13^ or National Institute for Health and Care Excellence (NICE) guidance (from 2007 onwards).^14^ These criteria are broadly similar, however, CDC criteria specify symptom duration of 6 months compared with 4 months in the NICE guidelines. CDC criteria also require that patients present with at least four of eight named symptoms (subjective memory impairment, sore throat, tender lymph nodes, muscle pain, joint pain, headaches, unrefreshing sleep and postexertional malaise), whereas NICE criteria include these symptoms (plus flu-like symptoms, dizziness, nausea and palpitations) for guidance only.

### Netherlands paediatric clinical cohort

Children were recruited from paediatricians throughout the Netherlands for the FITNET trial for adolescents with CFS/ME, between January 2008 and February 2010.^8^ Children were included in the trial if they were aged 12–18 years, had a primary diagnosis of CFS/ME in accordance with CDC criteria, had severe fatigue (Checklist Individual Strength (CIS20-R)^15^ subjective fatigue subscale score >40) and were physically disabled (Child Health Questionnaire (CHQ-CF87)^16^ physical function subscale score ≤85 or school attendance ≤85%). Children were assessed by a paediatrician and had screening blood tests to exclude other causes of fatigue.

### Ethical approvals

#### UK cohorts

The North Somerset and South Bristol Research Ethics Committee decided that collection and analysis of CFS/ME patient data constituted service evaluation and did not require ethical review by a NHS Research Ethics Committee or approval by NHS Research and Development offices (REC reference number 07/Q2006/48).

#### Dutch cohort

The FITNET study reviewed and approved by the Medical Ethical Committee of the University Medical Centre Utrecht (reference 07/196-K) and the Medical Ethical Committee of the Radboud University Nijmegen Medical Centre (reference AMO number 07/105). Patients and their parents will receive verbal and written information about the study and informed consent will be obtained before randomisation. Trial registration: ISRCTN59878666 and (ClinicalTrials.gov) NCT00893438.

### Clinical and patient-reported measures

#### Symptoms and comorbidities

In the UK and in the Netherlands, clinicians prospectively collected symptoms used to make a diagnosis of CFS/ME (cognitive dysfunction, sleep disturbance/unrefreshing sleep, postexertional malaise, joint pain, muscle pain, headaches, painful lymph nodes, sore throat, general malaise/flu-like symptoms, dizziness, nausea and palpitations).^14^ ^13^ In the UK, adult and paediatric services also prospectively asked patients whether they had received a diagnosis of depression or anxiety, migraine, irritable bowel syndrome, Fibromyalgia or Chronic Regional Pain Disorder. Time to assessment was calculated as the time from reported symptom onset to assessment in the CFS/ME specialist clinic.

#### Fatigue

In the UK, fatigue was measured using the Chalder fatigue scale (range 0–33).^17^ Internal consistency for this instrument, calculated by Cronbach’s α, ranges from 0.88 to 0.90.^18^ In the Netherlands, fatigue was measured using the ‘subjective fatigue’ subscale of the CIS20-R (range 8–56). This questionnaire has good reliability, discriminative validity and internal consistency (Cronbach's α=0.93).^19^

#### Disability

In the UK, disability was measured using the 10-item physical function subscale of the Short form 36 (SF-36).^20^ Children scored between 0 (‘yes, limited a lot’) and 10 (‘no, not limited at all’) for each item, so that children with the worst physical function scored 0 while those with good physical function scored 100. In the Netherlands, physical disability was measured with the physical functioning subscale of the CHQ-CF87.^16^ This is scored 0–100% with a lower score indicating more disability. This assessment tool is reliable and has been validated with a good internal consistency (Cronbach's α=0.86).^21^

#### School attendance

School attendance was measured in the UK by asking how the young person would describe their attendance as a percentage of expected attendance: ‘None’, ‘About 10% (eg, one half day)’, ‘About 20% (eg, one day)’, ‘About 40% (eg, two days)’, ‘About 60% (eg, three days)’, ‘About 80% (eg, four days)’ and ‘Full time (100%)’. In the Netherlands, school attendance was self-recorded daily for 12 days prior to assessment and averaged. These data were shown to be consistent with school records.^8^

#### Pain

In the UK, a visual analogue pain rating scale was used to measure pain prior to assessment, with a score of 0 for ‘no pain’ and 100 for ‘pain as bad as possible’. Pain was categorised as: none (0–4); mild (5–44), moderate (45–74), severe (75–100). In the FITNET trial, participants recorded their pain level using a Likert scale (0–4) at four separate times each day for 12 days prior to assessment.^22^

#### Mood

In the UK, the Hospital and Anxiety Depression Scale (HADS)^23^ was used to assess mood in children aged 12 years and older. The HADS is a 14-item inventory with anxiety and depression subscales.^24^ Cut-off scores for HADS depression were >9 for boys, >10 for girls^25^ and >12 for adults.^26^ Likewise, the cut-off for identifying clinical presentations of anxiety symptoms was >12 for boys, >16 for girls,^25^ >11 for men and >12 for women.^26^ In the Netherlands, anxiety was assessed using the Spielberger State-Trait Anxiety Inventory for Children (STAIC),^27^ comprising 20 questions, each on a 3-point scale. Depression was measured using a Dutch translation of the 27-item Children's Depression Inventory (CDI).^28^ This instrument has a high degree of internal consistency, with Cronbach's α ranging from 0.71 to 0.89.^29^ Cut-off scores for depression and anxiety were CDI score >15 and STAIC score >43.^8^

### Statistical analyses

We performed descriptive analyses separately for each of the four groups: UK children aged <12 years, UK adolescents aged 12–18 years, Dutch adolescents aged 12–18 years and UK adults (aged 19–65 years). We used linear regression to estimate differences in disability and fatigue between UK paediatric and adult patients. We used multinomial logistic regression analyses to compare child and adolescent with adult (reference category) groups, controlling for gender. Binary logistic regression analyses were used to test for differences between adolescents from the UK and the Netherlands. Inventories were coded as missing if >1 question was missing. On the HADS, each seven-item subscale was excluded if there was more than one question missing. Questions for which two answers were given were coded as missing. Total scores were corrected for the number of missing items. Stata (StataCorp 2013, Stata Statistical Software: Release 13, College Station, Texas, USA: StataCorp LP) was used for all analyses.

## Results

The UK paediatric cohort comprised 1568 UK adolescents aged 12–18 years and 210 children aged <12 years. Data on comorbid disorders were collected from 2010, and these data were available for approximately 900 children and adolescents (exact numbers shown in table 1). Body mass index (BMI) data were available for 37% (579/1568) of the adolescents and 24% (49/204) of the younger children. The UK adult cohort comprised 10 675 patients. Symptom data were collected from adults since 2010, and these data were available for approximately 7000 adults (exact numbers shown in table 1). The Dutch adolescent group had almost complete data, with missing data on BMI in only one patient.

**Table 1:** Differences in the presence of symptoms among UK children, adolescents and adults with CFS/ME, showing ORs derived from multinomial logistic regression controlling for gender (adults as reference group unless otherwise indicated)

Symptoms	Younger children (aged <12 years)		Adolescents (aged 12–18 years)		Adults (aged 19–65 years)
	N/total (%)	OR (95% CI)	N/total (%)	OR (95% CI)	N/total (%)
Cognitive dysfunction	156/204 (76.5)	0.18 (0.13 to 0.25)	1281/1478 (86.7)	0.34 (0.28 to 0.40)	6586/6921 (95.2)
Sore throat	127/204 (62.3)	1.42 (1.07 to 1.90)	860/1482 (58.0)	1.10 (0.98 to 1.24)	3834/6823 (56.1)
Tender lymph nodes	95/203 (46.8)	1.02 (0.77 to 1.36)	601/1469 (40.9)	0.74 (0.66 to 0.83)	3314/6785 (48.8)
Muscle pain	154/204 (75.5)	0.45 (0.32 to 0.63)	1093/1484 (73.7)	0.38 (0.33 to 0.43)	6133/6944 (88.3)
Joint pain	116/205 (56.6)	0.43 (0.32 to 0.57)	895/1483 (60.4)	0.47 (0.42 to 0.53)	5276/6878 (76.7)
Headaches	152/204 (74.5)	1.14 (0.83 to 1.57)	1206/1487 (81.1)	1.56 (1.36 to 1.80)	5063/6870 (73.7)
Sleep dysfunction	171/201 (85.1)	0.23 (0.15 to 0.34)	1394/1462 (95.4)	0.77 (0.59 to 1.02)	6678/6926 (96.4)
Postexertional malaise	196/205 (95.6)	0.50 (0.25 to 0.99)	1444/1485 (97.2)	0.75 (0.53 to 1.07)	6748/6890 (97.9)
Nausea	87/205 (42.4)	0.59 (0.44 to 0.78)	581/1478 (39.3)	0.56 (0.50 to 0.63)	3602/6796 (53.0)
Palpitations	32/113 (28.3)	0.71 (0.47 to 1.08)	329/985 (33.4)	0.85 (0.73 to 0.97)	2533/6771 (37.4)
Dizziness	86/201 (42.8)	1.05 (0.79 to 1.39)	451/1479 (30.5)	0.65 (0.57 to 0.73)	2735/6816 (40.1)
General malaise/flu-like symptoms	77/116 (66.4)	0.69 (0.47 to 1.02)	661/987 (67.0)	0.68 (0.59 to 0.78)	5131/6835 (75.1)
Pain					
No pain (ref cat)	18/180 (10.0)	–	166/1349 (12.3)	–	635/8779 (7.2)
Mild pain	51/180 (28.3)	0.69 (0.40 to 1.19)	370/1349 (27.4)	0.52 (0.43 to 0.64)	2731/8779 (31.1)
Moderate pain	73/180 (40.6)	0.84 (0.49 to 1.41)	538/1349 (39.9)	0.61 (0.50 to 0.74)	3436/8779 (39.1)
Severe pain	38/180 (21.1)	0.76 (0.43 to 1.35)	275/1349 (20.4)	0.54 (0.44 to 0.67)	1977/8779 (22.5)
Depression symptoms (HADS)	–	–	379/1360 (27.9)	1.51 (1.33 to 1.72)	1916/9420 (20.3)
Anxiety symptoms (HADS)	–	–	285/1357 (21.0)	0.46 (0.41 to 0.53)	3420/9393 (36.4)
Anxiety symptoms (SCAS)*	56/173 (11.3)	1.09 (0.75 to 1.58)	440/759 (58.0)	–	–
Comorbid disorders					
Depressive disorder	0/10 (0.0)	–	61/785 (7.8)	0.17 (0.13 to 0.23)	2195/4488 (32.8)
Anxiety disorder	9/99 (9.1)	0.19 (0.10 to 0.38)	128/782 (16.4)	0.37 (0.30 to 0.45)	2328/6655 (35.0)
Migraine	5/106 (4.7)	0.19 (0.08 to 0.46)	126/818 (15.4)	0.63 (0.52 to 0.77)	1533/6747 (22.7)
Irritable bowel syndrome	8/106 (7.6)	0.17 (0.08 to 0.35)	67/807 (8.3)	0.18 (0.14 to 0.23)	2312/6749 (34.3)
Fibromyalgia	2/105 (1.9)	0.05 (0.01 to 0.21)	23/813 (2.8)	0.07 (0.05 to 0.11)	1919/6707 (28.6)
Regional pain syndrome	2/105 (1.9)	0.50 (0.12 to 2.03)	12/808 (1.5)	0.37 (0.21 to 0.67)	261/6667 (3.9)

*Reference group: children aged 12–18 years.

CFS/ME, chronic fatigue syndrome/myalgic encephalomyelitis; HADS, Hospital and Anxiety Depression Scale; SCAS, Spence Children's Anxiety Scale.

Table 2 shows that younger children (<12 years old) had a more equal gender balance (56.7% female) compared to adolescents (74.1% and 82.2% in the UK and the Netherlands, respectively) and adults (77.9%). BMI was similar in younger children and UK and Dutch adolescents. Duration of illness prior to assessment was longest in adults (median 36 months), compared to 16 months in UK adolescents and 12 months in UK primary school-aged children. Disability and fatigue were similar in younger and older UK children, but adults had lower mean physical function than adolescents (–8.2 points (95% CI −9.7 to −6.7 points) on SF-36 physical function subscale) and higher mean fatigue (2.1 points (1.8–2.4 points) on the Chalder Fatigue Scale. Adjustment for duration of illness did not change the difference in fatigue, but did slightly attenuate the difference in physical function (−6.7 points (−8.2 to −5.2 points)). Mean disability scores were not directly comparable between UK and Dutch adolescents because these had been obtained using different instruments.

**Table 2:** Characteristics of UK children, adolescents and adults, and Dutch adolescents

Characteristics	UK (aged <12 years)		UK (aged 12–18 years)		NL (aged 12–18 years)		UK adults (aged 19–65 years)	
	N		N		N		N	
Age (years), mean (SD)	210	9.3 (2.0)	1568	15.0 (1.8)	135	15.8 (1.3)	10675	40.1 (11.7)
Sex (female), frequency (%)	210	119 (56.7)	1568	1162 (74.1)	135	111 (82.2)	10675	8 (77.9)
BMI (kg/m^2^), mean (SD)	49	18.5 (3.8)	579	22.1 (6.5)	134	21.0 (2.9)	5598	26.2 (5.7)
Duration of illness (months), median (IQR)	161	12, 7–23	1293	18, 11–28	135	16, 9–28	8488	36, 16–96
Disability*, median (IQR)	178	45, 25–65	1411	50, 33–70	135	52, 48–55	9282	40, 20–60
Fatigue† (Chalder), median (IQR)	190	23, 20–28	1425	25, 22–29	–	–	9405	28, 24–31

*UK cohorts used SF-36 physical function subscale to measure disability. The Dutch cohort used the CHQ-CF87 physical function subscale.

†All Dutch children had severe fatigue (CIS20-R subjective fatigue scale score ≥40).

BMI, body mass index; CHQ-CF87, Child Health Questionnaire; SF-36, short form 36.

### Symptoms and comorbidities in young children, adolescents and adults (UK cohorts)

Table 1 shows that children under 12 (76.5%; OR 0.18 (95% CI 0.13 to 0.25)) are less likely to describe cognitive symptoms at assessment compared to adults (95.3%) and adolescents (86.7%). They are also less likely to have problems with sleeping (85.1% vs 96.4%; OR 0.23 (0.15, 0.34)). They are more likely to present with sore throats (62.3%; OR 1.42 (1.07% to 1.90%)) compared to adults (56.1%). The symptom of postexertional malaise is slightly less common in those under 12 (95.6%; OR 0.50 (0.25% to 0.99%)) than in adults (97.9%).

Adolescents are more likely to present with headaches (81.1%; OR 1.56 (1.36% to 1.80%)) compared to younger children (74.5%) and adults (73.7%). Adolescents were less likely to describe cognitive symptoms (86.7%; OR=0.34 (0.28% to 0.40%)) compared to adults (95.2%). Tender lymph nodes, palpitations, dizziness and general malaise, and either mild, moderate or severe pain (compared to ‘no pain’), were less likely in teenagers than in adults.

We compared symptoms between younger children and adolescents. Although younger children were less likely to present with cognitive dysfunction (OR 0.53 (0.37 to 0.76)), problems with sleep (OR 0.29 (0.19 to 0.47)), or headaches (OR 0.73 (0.52 to 1.03)), they were more likely to present with recurrent frequent sore throats (OR 1.29 (0.95 to 1.75)), tender lymph nodes (OR 1.38 (1.03 to 1.86)) and dizziness (OR 1.62 (1.20 to 2.20)).

Adolescents were more likely to score above the HADS threshold for depression (27.9% vs 20.3%, OR 1.51 (1.33 to 1.72)), but below the threshold for anxiety (21%, OR 0.46 (0.41 to 0.53)), compared to adults (36.2%). However, previous diagnoses of depression and anxiety (recorded as comorbid by the clinician at time of assessment) were much less common in children and adolescents compared to adults, as were all of the other comorbidities (migraine, irritable bowel syndrome, fibromyalgia and regional pain syndrome).

### Comparison between adolescents in the UK and the Netherlands

Table 3 shows that teenagers (aged 12–18 years) with CFS/ME tended to present with similar symptoms in the UK and the Netherlands, but postexertional malaise (97.2% vs 86.7%; OR 5.81 (3.21 to 10.51)), severe pain (20.4% vs 9%; OR 3.12 (1.49 to 6.54)), depression (27.9% vs 17%; OR 2.01 (1.26 to 3.22) and anxiety (21% vs 11.1%; OR 2.62 (1.49 to 4.59)), were more common in UK adolescents, and headaches (81.1% vs 89.6%; OR 0.54 (0.30 to 0.96)) were less common.

**Table 3:** Differences in the presence of symptoms among UK and Dutch adolescents with CFS/ME, showing OR derived from logistic regression controlling for age and sex (Dutch adolescents as reference group)

		UK		Netherlands	
	N	Frequency (%)	N	Frequency (%)	OR (95% CI)*
Cognitive dysfunction	1478	1281 (86.7)	135	123 (91.1)	0.77 (0.41 to1.43)
Sore throat	1482	860 (58.0)	135	78 (57.8)	1.03 (0.72 to 1.48)
Tender lymph nodes	1469	601 (40.9)	135	49 (36.3)	1.28 (0.88 to 1.85)
Muscle pain	1484	1093 (73.7)	135	103 (76.3)	0.93 (0.61 to 1.41)
Joint pain	1483	895 (60.4)	135	87 (64.4)	0.86 (0.59 to 1.24)
Headaches	1487	1206 (81.1)	135	121 (89.6)	0.54 (0.30 to 0.96)
Sleep dysfunction	1462	1394 (95.4)	135	129 (95.6)	1.02 (0.43 to 2.42)
Postexertional malaise	1485	1444 (97.2)	135	117 (86.7)	5.66 (3.07 to 10.45)
Pain (ref no pain)†	1349	166 (12.3)	134	22 (16.4)	–
Mild pain	1349	370 (27.4)	134	56 (41.8)	0.98 (0.57 to 1.67)
Moderate pain	1349	538 (39.9)	134	44 (32.8)	1.81 (1.04 to 3.13)
Severe pain	1349	275 (20.4)	134	12 (9.0)	3.12 (1.49 to 6.54)
Depression‡	1360	379 (27.9)	135	23 (17.0)	2.01 (1.26 to 3.22)
Anxiety§	1357	285 (21.0)	135	15 (11.1)	2.62 (1.49 to 4.59)

*Adjusted for age and sex.

†Pain in UK adolescents was measured on a visual analogue scale, categorised as none (0–4), mild (5–44), moderate (45–74) and severe (75–100); pain in Dutch adolescents was measured on a 0–4 Likert scale at four separate times each day for 12 days.

‡Depression in UK adolescents was defined as HADS depression score >9 (boys) or >10 (girls); depression in Dutch adolescents was defined as CDI score >15.

§Anxiety in UK adolescents was defined as HADS anxiety score >12 (boys) or >16 (girls); anxiety in Dutch adolescents was defined as STAIC score >43.

CDI, Children's Depression Inventory; CFS/ME, chronic fatigue syndrome/myalgic encephalomyelitis; HADS, Hospital and Anxiety Depression Scale; STAIC, Spielberger State-Trait Anxiety Inventory for Children.

## Discussion

This is the first study to show that children with CFS/ME present differently compared to adults with CFS/ME. Children and adolescents had less fatigue and better physical function, and were much less likely to have been given a diagnosis of comorbid illnesses, including depression and anxiety. Younger children were more likely to present with sore throat and were less likely to have sleep or memory problems, often considered cardinal symptoms in adults. Adolescents were more likely to present with cognitive and sleep dysfunction than were younger children, but less likely to present with tender lymph nodes and dizziness. Despite differences in sampling, UK adolescents with CFS/ME were similar to teenagers in the Netherlands, suggesting that the symptomatology of paediatric CFS/ME is generalisable to other populations and is robust across different healthcare settings.

The main strengths of this study were the large sample sizes, and the fact that symptoms and comorbid mood disorders were collected in the same way in UK children and adults. Adults and children were recruited from specialist CFS/ME services in the UK and the Netherlands, so the results may not be generalisable to other settings, such as primary care. Fatigue, disability, pain and mood were measured differently in the UK and the Netherlands, making it difficult to directly compare these characteristics. Also, while there is evidence for the validity and reliability of these instruments for adults diagnosed with CFS/ME,^30^ their use among paediatric CFS/ME patients has not been thoroughly evaluated,^31^ and we cannot assume that scores for adults, adolescents and children are comparable.

Data for UK adolescents were collected routinely as this group attended specialist CFS/ME services, whereas data for Dutch adolescents were obtained from a clinical trial. In the Dutch cohort, CFS/ME was diagnosed or confirmed in a tertiary academic hospital setting. However, since referrals were obtained nationwide from various sources (general practitioners as well as paediatricians), we consider that our study population was representative of the Dutch paediatric CFS/ME population.

The differences in symptom presentation that we found between younger and older children were not found in our previous comparison of these age groups,^10^ probably because this earlier study was insufficiently powered to detect such differences. It is striking that the gender balance is more equal in younger children (57% female), compared to adolescents (74.1%) and adults (77.9%). This shift in gender balance is consistent with population data, where an equal gender balance (49.6% female) has been described in a population cohort at age 13 years.^32^ Studies recruiting teenagers from secondary schools have consistently shown a female-to-male ratio of approximately 3:1,^33^ ^34^ suggesting that, during adolescence, the prevalence of CFS/ME increases in females but not in males.^35^

Adolescents from the UK and the Netherlands presented with similar symptoms, although headaches were less common, and postexertional malaise, severe pain and mood disorders were more common in UK patients. The difference in postexertional malaise could be explained by the fact that this is considered a cardinal symptom in the UK. The lower prevalence of anxiety and depression in the Dutch cohort could be because high scores for mood disorder were a reason for further psychological assessment to exclude primary mood disorders, leading to possible exclusion from the study. Also, the UK and Dutch cohorts used different instruments to measure mood (and pain), and this is likely to contribute to discrepancies when these measures are categorised to indicate presence/absence (or degree) of symptoms.

Paediatricians need to be aware that children with CFS/ME present differently from adults with CFS/ME. Younger children are more likely to present with recurrent sore throats and teenagers are likely to present with headaches. Further research should investigate whether these differences in presentation reflect the underlying aetiopathology or explain prognostic differences.
