# Correction: *Chronic fatigue syndrome (CFS) or myalgic encephalomyelitis (ME) is different in children compared to in adults: a study of UK and Dutch clinical cohorts*

**DOI:** 10.1136/bmjopen-2015-008830corr1

**Published:** 2019-11-20

Collin SM, Nuevo R, van de Putte EM, *et al*. Chronic fatigue syndrome (CFS) or myalgic encephalomyelitis (ME) is different in children compared to in adults: a study of UK and Dutch clinical cohorts. *BMJ Open* 2015;5:e008830. doi: 10.1136/bmjopen-2015-008830

The “Ethics approval” statement in this article has been amended to read:Ethics approval: The patient data used in this study were collected as part of routine clinical practice and anonymised for the National Outcomes Database. Under the Governance Arrangements for Research Ethics Committees {September 2011), ethical review is not required for research limited to the use of previously collected, non-identifiable patient information.

In addition, the following two sentences have been amended.

- The second paragraph under Strengths and limitations should read “Adults and children were from specialist CFS/ME services in the UK and the Netherlands, so the results may not be generalisable to other settings, such as primary care.
- The second sentence of second paragraph under Discussion should read “Adults and children were from specialist CFS/ME services in the UK and the Netherlands, so the results may not be generalisable to other settings, such as primary care.”

